# “Mini Molar Tooth” Sign in *POLR3B* ‐Associated Cerebellar Ataxia with Hypomyelinating Leukodystrophy

**DOI:** 10.1002/mdc3.70176

**Published:** 2025-06-11

**Authors:** Luca Marsili, Marcelo A. Kauffman, Blanca Talavera de la Esperanza, Zheming Yu, Donald L. Gilbert, Alberto J. Espay

**Affiliations:** ^1^ James J. and Joan A. Gardner Center for Parkinson's Disease and Movement Disorders, Department of Neurology University of Cincinnati Cincinnati Ohio USA; ^2^ Consultorio y Laboratorio de Neurogenética, Centro Universitario de Neurología José María Ramos Mejía Buenos Aires Argentina; ^3^ Division of Neurology Cincinnati Children's Hospital Medical Center Cincinnati Ohio USA; ^4^ Department of Pediatrics University of Cincinnati College of Medicine Cincinnati Ohio USA

**Keywords:** POLR3B, ataxia, hypomyelinating leukodystrophy, molar tooth sign

In 2003, we reported two ataxic sisters with hypomyelinating leukodystrophy within the spectrum of the cerebello‐cerebral‐oculo‐renal syndrome but with negative genetic workup.^1^ Two decades later, whole‐exome sequencing (WES) revealed pathogenic heterozygous variants in *POLR3B*. Besides resolving a previously undiagnosed but well characterized disorder, we aim to highlight what we refer to as the “mini molar tooth” sign, originally suggesting a variant of Joubert syndrome. We propose that this imaging sign is a diagnostic clue in *POLR3B*‐related leukodystrophy.

Both sisters, first evaluated in their 20s, exhibited developmental delay, ataxia, hypogonadism, and poorly formed secondary teeth. Neurologic examination showed impaired eye abduction, dysarthria, bradylalia, hypoplastic optic nerves, jerky smooth pursuit, slow initiation of saccades, horizontal nystagmus, and appendicular and truncal ataxia. Both had hypoplastic, discolored teeth, and required pharmacologic induction and maintenance of menstruation.

No longer progressing after late adolescence, the sisters are now in their late forties (Video 1 and 2).

**Video 1:** Features of dysarthria and speech and cerebellar ataxia of Sister 1 (now 49 years).

**Video 2:** Features of dysarthria and speech and cerebellar ataxia of the more affected younger Sister 2 (now 47 years).

Imaging findings showed absent myelination in the centrum ovale and corpus callosum, anterior commissure, and U‐fibers except for a single bundle in one sister.^1^ The severely hypoplastic cerebellum, leaving behind a remnant of the anterior vermis along with hypoplasia of the superior cerebellar peduncles, yielded a “mini molar tooth” sign, reminiscent of the “molar tooth sign” of Joubert syndrome (Fig. 1, second image).

**Figure 1:**
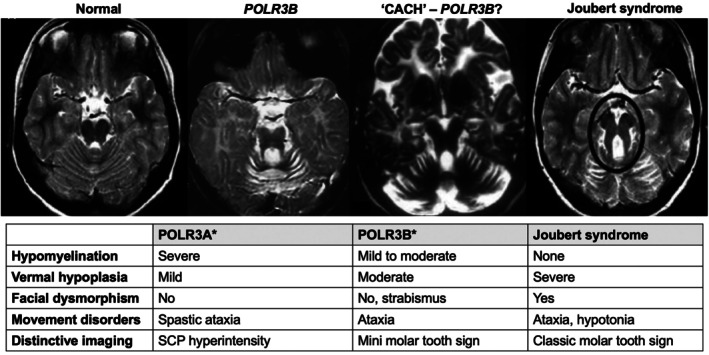
From “mini‐molar tooth” to classic “molar tooth” sign. Axial T2‐weighted brain MRIs showing, from left to right, normal midbrain; *POLR3B*‐associated ataxia (sister 1 at 19 years of age as originally reported), childhood ataxia with cerebral hypomyelination (CACH, undiagnosed case report of three young siblings most likely associated with a *POLR3B* mutation), and a the classic “molar tooth” sign (within black circle), typical of Joubert syndrome. Compared with the classic molar tooth sign of Joubert syndrome, the “mini molar tooth” sign exhibits thin rather than thick superior cerebellar peduncles and a relatively shallow rather than deep anterior foramen cecum. * Both POLR disorders yield variants of an “4H” imaging phenotype: Hypomyelination, hypodontia, and hypogonadotropic hypogonadism. SCP: Superior cerebellar peduncle. Permission was granted to republish second^1^, third^2^ and fourth MRI^3^.

WES analysis of Sister 2 identified two heterozygous pathogenic variants in *POLR3B* (OMIM614366) [**Variant 1:** *NM_018082.6:c.1568 T>A*(p.Val523Glu), pathogenic based on criteria PM2, PP3, PS1; **Variant 2:** *NM_018082.6:c.3082C>T*(p.Arg1028Ter), a presumed novel nonsense variant, pathogenic based on criteria PVS1, PM2, PP3]. The first variant has been previously reported; the second variant, not previously documented, is consistent with loss‐of‐function and meets established pathogenicity criteria. The presence of these two pathogenic variants supports a molecular diagnosis of *POLR3B*‐related leukodystrophy (OMIM614381).

The “molar tooth” sign is characterized by cerebellar vermis hypoplasia with enlarged fourth ventricle, increased depth of the interpeduncular fossa, and elongated/reoriented superior cerebellar peduncles. It is a signature feature of Joubert syndrome, which includes intellectual disability, episodic hyperpnea, abnormal eye movements, ataxia, although it can be associated with other syndromes, including Dekaban‐Arima, Senior‐Löken, COACH (cerebellar vermis hypoplasia, oligophrenia, ataxia, coloboma, hepatic fibrosis).^4^ Neither Joubert nor any of these other developmental abnormalities exhibit hypomyelination. The difference of the midbrain/vermis configuration with regards to the molar tooth sign on imaging between the *POLR3B*‐ataxia sisters and Joubert syndrome is a thin rather than thick superior cerebellar peduncles and shallower anterior foramen cecum. We propose that this ‘mini molar tooth’ sign be considered an imaging clue for underlying *POLR3B* mutation. A strikingly similar finding (Fig. 1, third image) was reported in three siblings with onset at 5 years of cerebellar ataxia with cerebral hypomyelination, which may have been the result of a *POLR3B* mutation (the corresponding author of this publication indicated he no longer can reach these siblings).^2^

*POLR3*‐related leukodystrophy includes several phenotypes associated with ataxia: hypomyelination, hypodontia, hypogonadotropic hypogonadism (4H syndrome); delayed dentition, and hypomyelination; tremor‐ataxia with central hypomyelination; leukodystrophy with oligodontia, and hypomyelination with cerebellar atrophy and hypoplasia of the corpus callosum.^2^, ^5^ *POLR3A* encodes the largest subunit of RNA polymerase‐III (Pol‐III), and *POLR3B* the second largest Pol‐III subunit.^6^ *POLR3A* mutations are associated with earlier, more severe clinical and hypomyelinating phenotype, and a shorter life expectancy, whereas *POLR3B* mutations with milder symptoms and less aggressive disease course but with more prominent cerebellar abnormalities on imaging. We suggest that if brain hypomyelination occur with hindbrain junction abnormalities suggestive of the “mini molar tooth” sign, *PORL3B* should be considered the underlying genotype.

## Authors’ Roles

(1) Research project: A. Conception, B. Organization, C. Execution; (2) Statistical Analysis: A. Design, B. Execution, C. Review and Critique; (3) Manuscript: A. Writing of the first draft, B. Review and Critique. A. Drafting of the case report; B. acquisition of data; C. analysis and interpretation; D. critical revision of the manuscript for important intellectual content.

L.M.: 1A, B, C, 3A.

M.A.K.: 3A, B.

B.T.D.L.E.: 1C,3B.

Z.Y.: 1C,3B.

D.L.G.: 3B.

A.J.E.: 1A, B, C, 3A, B.

All the co‐authors listed above gave their final approval of this manuscript version.

## Disclosure

**Ethical Compliance Statement:** This work did not require informed patient consent or the approval of an institutional review board. We confirm that we have read the Journal's position on issues involved in ethical publication and affirm that this work is consistent with those guidelines.

**Funding sources and conflict of interest:** The authors declare no funding sources or conflicts of interest relevant to this work.

**Financial disclusures for the previous 12 months:** LM has received honoraria from the International Association of Parkinsonism and Related Disorders (IAPRD) Society for social media and web support. LM has received a grant (collaborative research agreement) from the International Parkinson and Movement Disorders Society for the MDS‐UTRS Validation Program (Role: PI), Non‐Profit. MAK is an employee of the CONICET and has received grant support from the Ministry of Science and Technology of Argentina and the Ministry of Health of Buenos Aires. BTdlE has nothing to disclose. ZY has nothing to disclose. DLG has nothing to disclose. AJE has received grant support from the NIH and the Michael J. Fox Foundation; personal compensation as a consultant/scientific advisory board member for Mitsubishi Tanabe Pharma America (formerly, Neuroderm), Amneal, Acorda, Bial, Kyowa Kirin, Supernus (formerly, USWorldMeds), NeuroDiagnostics, Inc (SYNAPS Dx), Intrance Medical Systems, Inc., Merz, Praxis Precision Medicines, Citrus Health, and Herantis Pharma; Data Safety Monitoring Board (chair) of AskBio; and publishing royalties from Lippincott Williams & Wilkins, Cambridge University Press, and Springer. He is co‐inventor of the patent “Compositions and methods for treatment and/or prophylaxis of proteinopathies” and cofounded REGAIN Therapeutics to fund preclinical studies. He has relinquished his right to any personal income from future treatments.

## Data Availability

AJE had full access to all the data in the study and takes responsibility for the integrity of the data, the accuracy of the data analysis, and the conduct of the research. He has the right to publish any and all data, separate and apart from the guidance of any sponsor.
